# Renal protective effect of sacubitril/valsartan in patients with heart failure

**DOI:** 10.1038/s41598-021-84118-8

**Published:** 2021-02-25

**Authors:** Hui-Ling Hsieh, Chun-You Chen, Cheng-Hsien Chen, Shih-Chang Hsu, Wen-Cheng Huang, Yuh-Mou Sue, Feng-Yen Lin, Chun-Ming Shih, Yue-Cune Chang, Po-Hsun Huang, Chung-Te Liu

**Affiliations:** 1 Division of Nephrology, Department of Internal Medicine, Wan Fang Hospital, Taipei Medical University, Taipei, Taiwan; 2 Graduate Institute of Medical Science, National Defense Medical Center, Taipei, Taiwan; 3 Department of Radiation Oncology, Wan Fang Hospital, Taipei Medical University, Taipei, Taiwan; 4 Division of Nephrology, Department of Internal Medicine, Shuang Ho Hospital, Taipei Medical University, New Taipei City, Taiwan; 5 Department of Internal Medicine, School of Medicine, College of Medicine, Taipei Medical University, Taipei, Taiwan; 6 Emergency Department, Department of Emergency and Critical Medicine, Wan Fang Hospital, Taipei Medical University, Taipei, Taiwan; 7 Department of Emergency Medicine, School of Medicine, College of Medicine, Taipei Medical University, Taipei, Taiwan; 8 Graduate Institute of Clinical Medicine, College of Medicine, Taipei Medical University, Taipei, Taiwan; 9 Division of Cardiology and Cardiovascular Research Center, Department of Internal Medicine, Taipei Medical University Hospital, Taipei, Taiwan; 10 Department of Mathematics, Tamkang University, New Taipei City, Taiwan; 11 Division of Cardiology, Department of Medicine, Taipei Veterans General Hospital, Taipei, Taiwan; 12 Cardiovascular Research Center, National Yang-Ming University, Taipei, Taiwan; 13 Institute of Clinical Medicine, National Yang-Ming University, Taipei, Taiwan

**Keywords:** Cardiology, Diseases, Nephrology

## Abstract

Sacubitril/valsartan is a combined neprilysin inhibitor/angiotensin II receptor blocker designed for treatment of heart failure (HF). Nonetheless, its renal protective effect remained an issue of debate. This retrospective cohort study investigated the renal protective effect of sacubitril/valsartan in HF patients. HF patients on sacubitril/valsartan or valsartan for > 30 days were matched for gender, age, estimated glomerular filtration rate (eGFR), and left ventricular ejection fraction (LVEF) to be enrolled into analysis. The follow-up period was 18 months. The outcomes included end eGFR, renal function decline defined as 20% reduction of eGFR, mortality, and HF-related hospitalization. Each group had 137 patients after matching. The mean age was 72.7 years and 65.7% were male. Mean eGFR was 70.9 mL/min/1.73 m^2^ and LVEF was 54.0% at baseline. Overall, the eGFR of sacubitril/valsartan groups was significantly higher than valsartan group at the end (*P* < 0.01). Subgroup analysis showed that the difference in eGFR was significant in subgroups with LVEF ≥ 40% or eGFR ≥ 60 mL/min/1.73 m^2^. Multivariate Cox regression model showed that sacubitril/valsartan group had significantly reduced risk for renal function decline (hazard ratio: 0.5, 95% confidence interval: 0.3–0.9). Kaplan–Meier curve showed no difference in the risk for cardiovascular mortality, all-cause mortality or HF-related hospitalization. We showed renal protective effect of neprilysin inhibition in HF patients and specified that subgroups with LVEF ≥ 40% or eGFR ≥ 60 mL/min/1.73 m^2^ were sensitive to this effect, suggesting an optimal subgroup of this treatment.

## Introduction

Heart failure (HF) affects as many as 26 million patients worldwide and has become an important global health issue^1,2^. In U.S.A., the prevalence of HF is predicted to increase from 2.42% in 2012 to 2.97% in 2030, resulting an annual cost of 69.8 billion U.S. dollar^3^. In a report released by National Health Insurance of Taiwan, over 22,000 people out of the total 2.3 million population in Taiwan had been hospitalized due to HF in 2014^4^. These reports suggest a huge HF-related medical burden. Another study conducted in U.S.A. showed that after the diagnosis of HF, 83.1% of patients had been hospitalized for at least once. Notably, in this report, only 16.5% of hospitalization were due to HF, and 61.9% were non-cardiovascular hospitalization, showing an important role of noncardiac comorbidities in HF patients^5^.

In patients with HF, the presence of chronic kidney disease (CKD) had been shown to predict all-cause hospitalization, suggesting an interaction between the dysfunction of these two vital organs^5–8^. The conception of cardiorenal syndrome (CRS) had been proposed in 2008, which include 5 types classified by different pathophysiology. Among all types, type 2 CRS comprises chronic HF causing progression of CKD, which meets the scenario of a large part of patients with combined HF and CKD^9^. Over the last decade, the classification, pathophysiology, diagnosis, and treatment strategies of CRS have been better acknowledged. Currently, the mainstay of treatment for CRS was blockade of renin–angiotensin–aldosterone axis, which was based on renal outcomes of a large body of research^10^. However, treatment specifically targeting HF-related CKD remains absent to date.

Sacubitril/valsartan is a combined neprilysin inhibitor/angiotensin II receptor blocker (ARB), which prolongs the beneficial effects of natriuretic peptides and blocks the adverse effect from angiotensin II accumulation^11–14^. Recently, clinical trials showed that sacubitril/valsartan was superior to angiotensin converting enzyme inhibitor (ACEI) in improving outcomes of HF patients with reduced ejection fraction (HFrEF) (PARADIGM-HF trial)^15^ and lowering N-terminal pro-B-type natriuretic peptide in patients hospitalized due to acute decompensated HF^16^. By the attention, in PARADIGM-HF trial, the sacubitril/valsartan group exhibited significantly fewer events of renal function decline, implying that neprilysin inhibition may have renal protective effect^15^. Nonetheless, in PARADIGM-HF trial, the control group was treated with ACEI and the renal outcomes between the two groups may be biased from the differential effect of ARB and ACEI. Moreover, a similarly designed trial recruiting HF patients with median estimated glomerular filtration rate (eGFR) of 58 mL/min/1.73 m^2^ showed insignificant difference on renal outcomes, which argues that pre-existing renal impairment may confound renal effect of sacubitril/valsartan^17^. As such, to disclose the renal effects of neprilysin inhibition, a study should be designed to compare the difference between sacubitril/valsartan and valsartan with stratification by the presence of pre-existing renal impairment.

In this study, we hypothesized that sacubitril/valsartan has additional renal protective effect compared with valsartan in patients with subgroups of specific range of renal function. To that end, we conducted a retrospective, longitudinal cohort study to enroll HF patients on either sacubitril/valsartan or valsartan. The renal outcomes will be analyzed in subgroups stratified by baseline eGFR to identify patients susceptible to the renal protective effect of neprilysin inhibition.

## Materials and methods

### Study design and subjects

This study was conducted in Wanfang Hospital, Taipei Medical University between January 1, 2016 and Oct 31, 2018. Patients from outpatient department that fulfilled the following 3 criteria were included into the matching procedure: (1) Diagnosis of HF made by the attending cardiologist as documented on medical records; (2) Age of ≥ 20 years old; (3) Use of sacubitril/valsartan or valsartan for more than 30 days during the study period. The diagnosis of HF was based on 2019 Focused Update of the Guidelines of the Taiwan Society of Cardiology for the Diagnosis and Treatment of HF. HF is suspected in patients with either of the following findings: (1) typical symptoms or signs of HF (e.g. fatigue, dyspnea, peripheral edema) (2) pulmonary edema or cardiomegaly shown by chest x-ray file (3) T-inversion or ST-depression shown by electrocardiogram. Suspected patient with HF then underwent a test of plasma NT-proBNP. Plasma NT-proBNP of ≦300 pg/mL excludes the diagnosis of HF. Patients diagnosed with HF then received echocardiography for measurement of left ventricular ejection fraction (LVEF)^18^. The decisions of using sacubitril/valsartan were according to discretion of attending physicians based on individual requirement for treatment HF regardless of insurance reimbursement criteria. Eligible patients underwent data collection required for matching procedure, including gender, age, baseline eGFR, and LVEF measured by echocardiography. This matching procedure aimed to find two groups of sacubitril/valsartan and valsartan users with similar severity of HF and renal function at baseline. Patients with missing values, end-stage renal disease (ESRD) at baseline, and matching failure were excluded. Final data collection was then conducted in patients undergone successful matching process for an 18-month period since the initiation of sacubitril/valsartan or valsartan to assess the predictors and outcomes. Medications other than sacubitril/valsartan and valsartan were not controlled. This study was approved by the ethics committee and Institutional Review Board of Taipei Medical University (N202005099) and the informed consent was waived. The present study was also conducted in accordance with the tenets of the 1975 Declaration of Helsinki, as revised in 2000.

### Definitions of the covariates and outcomes

Baseline demographic profiles, laboratory data and LVEF measurements were defined as values obtained within 3 months before the initiation of sacubitril/valsartan or valsartan treatment. The definition and classification of HF was adapted from 2019 Focused Update of the Guidelines of the Taiwan Society of Cardiology for the Diagnosis and Treatment of Heart Failure^18^. In brief, patients with typical symptoms or signs of HF, NT-proBNP > 300 pg/mL and echocardiographic LVEF < 40% were defined as HFrEF; patients with typical symptoms or signs of HF, echocardiographic LVEF ≥ 40%, and key echocardiographic alterations of HF were defined as HF with preserved ejection fraction (HFpEF)^19^. Both types were considered eligible HF in the present study.

Other comorbidities, including diabetes mellitus (DM), hypertension, and atrial fibrillation (AFib) were defined according to the diagnosis documented in medical records. Notably, both paroxysmal and persistent AFib were considered equally as AFib in the present study. Patients who completed the matching process were followed for serum creatinine level, eGFR, and LVEF at 3, 6, 12, and 18 months. In the present study, echocardiographic LVEF was measured by attending cardiologist and eGFR was calculated by the equation suggested by the Chronic Kidney Disease Epidemiology Collaboration in 2009^20^.

The end point of the follow-up included discontinuation of sacubitril/valsartan or valsartan, and death from any cause. Patients reaching end points before 18 months of follow-up were censored in statistics involving survival analyses. The primary outcome of the present study was renal function decline, defined as reduction of eGFR ≥ 20% at any time during follow-up period. Notably, any renal function decline that resolved in 3 months were defined as acute kidney injury and were not counted as outcome events. The secondary outcomes included difference in eGFR, LVEF, hemoglobin and serum potassium levels between the two groups, death from cardiovascular causes, death from any causes, and HF-related hospitalization. Cause of death and hospitalization was based on the diagnosis of medical records of the indexed discharge summary.

### Statistical analysis

Continuous variables with normal distribution were presented as a mean ± standard deviation, and continuous variables deviated from a normal distribution were presented as the median and interquartile range. Statistical analyses for continuous variables with normal distribution were conducted using a two-tailed t-test for independent samples. Statistical analysis for difference of a single sample at two time points were conducted by using t-test for dependent samples. For continuous variables deviated from normal distribution, Wilcoxon-Mann–Whitney two-sample tests was used. Normality of data distribution was tested by using Kolmogorov–Smirnov test, in which *P* value > 0.05 indicated normal distribution. Categorical variables were presented as frequency and percentage. Statistical analyses of categorical variables were performed by using chi-square test.

The variables involving repeated measurements, including eGFR, LVEF, hemoglobin, and serum potassium levels measured at 3, 6, 12, and 18 months were analyzed by using generalized estimating equation (GEE)^21,22^. In analysis involving GEE, missing data were managed by default setting of statistical software. Notably, missing data were completely at random and thus did not bias the model^23^.

Analysis for renal decline events was conducted by using Cox proportional regression model, in which the association between predictors and outcomes were expressed as hazard ratio (HR) with 95% confidence interval (CI). Analyses for cardiovascular mortality, all-cause mortality and HF-related hospitalization were conducted by using Kaplan–Meier curves with log-rank test. *P* values < 0.05 were considered significant. Statistical analysis was performed using SAS 9.4 (SAS Institute Inc, Cary, NC, U.S.A.).

In order to assess the possibility of remaining confounding factors and improve the internal validity of the nonrandomized study, falsification analysis was performed^24,25^. In our study, we assessed the events of cancer, pneumonia, and fractures as falsification outcomes, which had no biologically plausible effect on reduction of eGFR, death from cardiovascular causes, death from any causes and HF-related hospitalization.

### Patient and public involvement

This research was done without patient involvement.

### Ethics approval

This manuscript is original and has not been considered for publication or published elsewhere in any form or language. The data and text in this manuscript are presented completely without split. The results are presented clearly, honestly, and without fabrication, falsification or inappropriate data manipulation.

## Results

### Baseline characteristics

During the study period, 227 patients prescribed with sacubitril/valsartan and 23,945 patients prescribed with valsartan fulfilling the inclusion criteria were enrolled for matching. After excluding patients with missing values required for matching, 221 patients in sacubitril/valsartan group and 14,030 patients in valsartan group remained to undergo matching procedure (Supplemental Table 1). After matching for gender, age, eGFR, and LVEF, 173 patients were selected into each group. After excluding patients with missing data and ESRD at baseline, 137 patients remained in each group for follow-up of the outcomes (Supplemental Fig. 1).

**Table 1:** Baseline demographic and laboratory characteristics.

Characteristics	Total	Sacubitril/valsartan	Valsartan	*P* value
	n = 274	n = 137	n = 137	
Male, n(%)	180(65.7)	90(65.7)	90(65.7)	1.00
Age, years	72.7 ± 14.9	72.7 ± 14.9	72.7 ± 14.9	0.98
DM, n(%)	116(42.3)	51(37.2)	65(47.5)	0.89
Hypertension, n(%)	274(100)	137(100)	137(100)	1.00
AFib, n(%)	54(19.7)	34(24.8)	20(14.6)	0.03
HF-related hospitalization, n(%)	49(17.9)	40(29.2)	9(6.6)	< 0.01
BUN, mg/dL	22(12)	22(12)	21(13)	0.52
Cr, mg/dL	1.0(0.4)	1.0(0.4)	1.0(0.5)	0.85
eGFR, mL/min/1.73 m^2^	70.9 ± 24.7	70.9 ± 24.6	71.0 ± 24.9	0.97
**eGFR groups, n**				
≥ 60	190	95	95	n/a
< 60 to ≥ 30 to	72	36	36	n/a
< 30	12	6	6	n/a
AST, U/L	20(10)	20(7)	20(9)	0.30
ALT, U/L	18(12)	18(12)	18(12)	0.85
Hemoglobin, g/dL	12.8(2.9)	12.9(2.8)	12.7(2.9)	0.97
K, mmol/L	4.2(0.7)	4.2(0.6)	4.1(0.8)	0.01
WBC, 10^3^/uL	7.7 (3.7)	7.2(3.8)	7.7(4.0)	0.06

*DM* diabetes mellitus, *AFib* atrial fibrillation, *HF* heart failure, *BUN* blood urea nitrogen, *Cr* creatinine, *eGFR* estimated glomerular filtration rate, *AST* aspartate transaminase, *ALT* alanine transaminase, *WBC*, white blood cell.

Among the included patients, the mean age was 72.7 ± 14.9 years old and 65.7% were male. Mean eGFR was 70.9 ± 24.7 mL/min/1.73 m^2^ and LVEF was 54.0 ± 15.7% at baseline. The portions of DM or hypertension were not significantly different between the two groups. The sacubitril/valsartan group had significantly more patients with AFib and more previous HF-related hospitalization. Baseline eGFR, and serum creatinine were not significantly different between the two groups (Table 1). Regarding echocardiographic measurements, sacubitril/valsartan group had slightly higher left ventricular end-systolic diameter than valsartan group. Otherwise, LVEF, relative wall thickness, left ventricular mass index along with other echocardiographic measurements, along with the frequency of valvular defects were not significantly different between the two groups (Table 2). The use of β-blockers, nitroglycerin, amiodarone, aspirin, clopidogrel, rivaroxaban, febuxostat, statin, and ivabradine were significantly more in sacubitril/valsartan group (Table 3). Although we had matched LVEF in attempt to include patients with similar severity of HF, regarding the difference in AFib patients, previous HF-related hospitalization, and medication profile, patients of sacubitril/valsartan group may have higher severity of HF compared with patients of valsartan group.

**Table 2:** Baseline echocardiographic profile.

Characteristics	Total	Sacubitril/valsartan	Valsartan	*P* value
	n = 274	n = 137	n = 137	
IVS, mm	13(3)	13(3)	13(3)	0.75
LVEDD, mm	51(11)	52(11)	50(11)	0.16
LVPW, mm	13(3)	13(2)	12(3)	0.47
LV mass, g	323.4(151.8)	325(154.9)	319.5(146)	0.26
LVEF, %	54.0 ± 15.7	52.7 ± 15.7	55.4 ± 15.6	0.15
LVESD, mm	35(16)	37(16)	34(14)	0.04
RWT, cm	0.50(0.13)	0.49(0.15)	0.50(0.11)	0.25
LVMI, g/m^2^	194.5(84.0)	200.1(84.0)	193.4(85.7)	0.30
BSA, m^2^	1.63 ± 0.17	1.63 ± 0.17	1.62 ± 0.16	0.57
MS, n(%)	0	0	0	n/a
MR, n(%)	75(27.4)	41(29.9)	34(24.8)	0.42
AS, n(%)	6(2.2)	3(2.2)	3(2.2)	1.00
AR, n(%)	38 (13.9)	19(13.9)	19(13.9)	1.00

*IVS* interventricular septum, *LVEDD* left ventricular end-diastolic diameter, *LVPW* left ventricular posterior wall, *LV mass* left ventricular mass, *LVEF* left ventricular ejection fraction, *LVESD* left ventricular end-systolic diameter, *RWT* relative wall thickness, *LVMI* left ventricular mass index, *BSA* body surface area, *MS* mitral stenosis, *MR* mitral regurgitation, *AS* aortic stenosis, *AR* aortic regurgitation.

**Table 3:** Baseline medication profiles of the cohort.

Characteristics	Total	Sacubitril/valsartan	Valsartan	*P* value
	n = 274	n = 137	n = 137	
Beta blockers, n(%)	131(47.8)	111(81.0)	20(14.6)	< 0.01
Dihydropiridine CCB, n(%)	47(17.2)	25(18.3)	22(16.1)	0.63
Non-dihydropiridine CCB, n(%)	10(3.7)	6(4.4)	4(2.9)	0.52
NTG, n(%)	22(8.0)	19(13.9)	3(2.2)	< 0.01
Amiodarone, n(%)	26(9.5)	24(17.5)	2(1.5)	< 0.01
Aspirin, n(%)	69(25.2)	52(38.0)	17(12.4)	< 0.01
Clopidogrel, n(%)	60(21.9)	55(40.2)	5(3.7)	< 0.01
Rivaroxaban, n(%)	29(10.6)	26(19.0)	3(2.2)	< 0.01
Warfarin, n(%)	6(2.2)	3(2.2)	3(2.2)	1.00
Febuxostat, n(%)	40(14.6)	36(26.3)	4(2.9)	< 0.01
Fibrates, n(%)	9(3.3)	7(5.2)	2(1.5)	0.17
Statin, n(%)	106(38.7)	88(64.2)	18(13.1)	< 0.01
Ivabradine, n(%)	17(6.2)	17(12.4)	0	< 0.01

*CCB* calcium channel blocker, *NTG* nitroglycerin.

### Effect of sacubitril/valsartan on eGFR relative to valsartan

**Figure 1:**
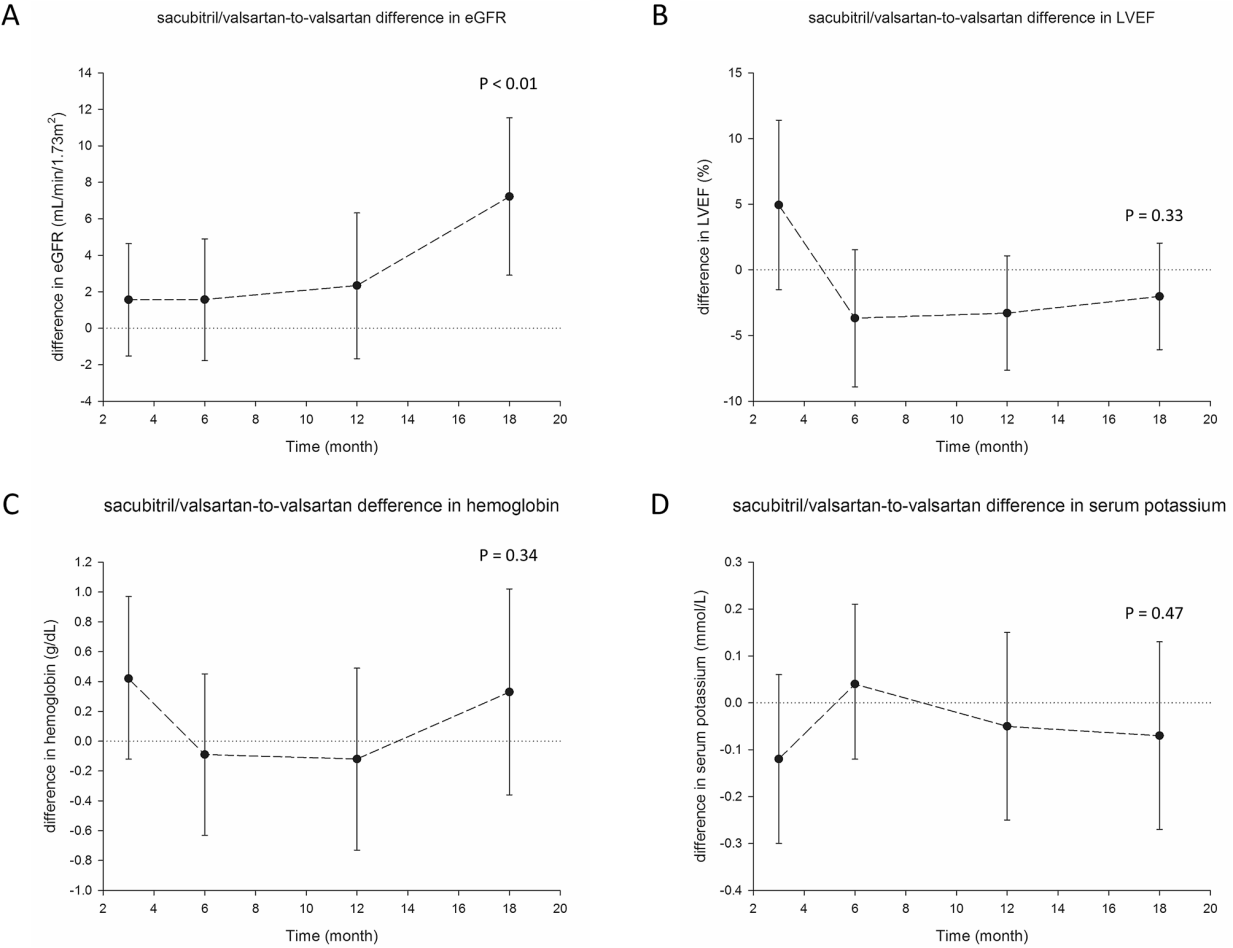
Difference of laboratory measurements and LVEF between patients on sacubitril/valsartan and valsartan. Difference of eGFR was calculated as sacubitril/valsartan group–valsartan group. eGFR, estimated glomerular filtration rate; LVEF, left ventricular ejection fraction. *P* value calculated by generalized estimating equation.

As included patients had been matched, the eGFR values of the two groups were equal at baseline (70.9 ± 24.7 mL/min/1.73 m^2^). After 18 months of follow-up, eGFR decreased to 69.4 mL/min/1.73 m^2^ in sacubitril/valsartan group and 63.9 mL/min/1.73 m^2^ in valsartan group, respectively. The decrease of eGFR in both groups were significant compared with respective baseline values (*P* < 0.01, Supplemental Fig. 2). The difference in eGFR between the two groups were analyzed by using GEE, which became significant at 18 months, showing that eGFR of sacubitril/valsartan group was 7.2 (95% CI: 2.9–11.5) mL/min/1.72 m^2^ higher than that of valsartan group (*P* < 0.01, Fig. 1A). The difference in LVEF, hemoglobin, and serum K level between the two groups were not significant throughout the study period (Fig. 1B–D).

**Figure 2:**
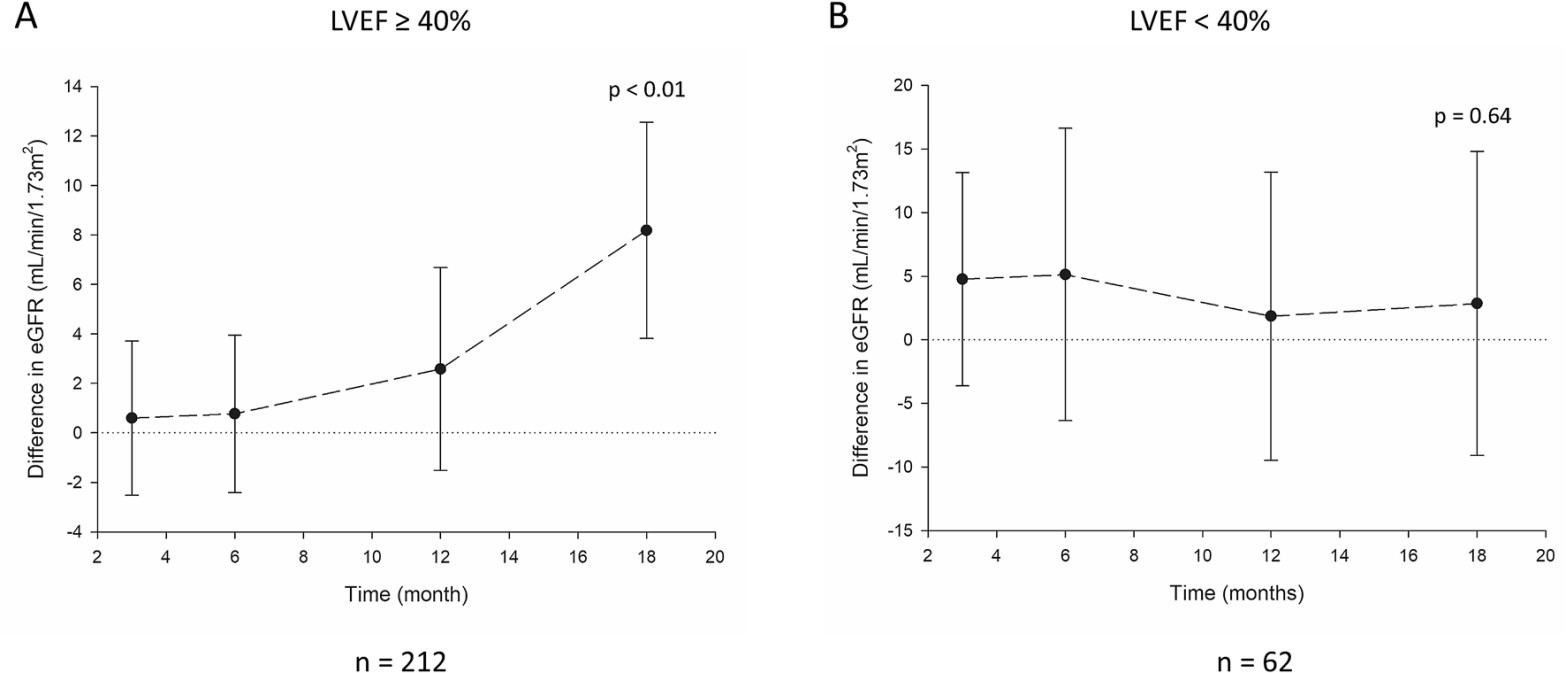
Difference of eGFR between patients on sacubitril/valsartan and valsartan stratified by LVEF. (**A**) LVEF ≥ 40% (**B**) LVEF < 40%. Difference of eGFR was calculated as sacubitril/valsartan group–valsartan group. eGFR, estimated glomerular filtration rate. *P* value calculated by generalized estimating equation.

Difference in eGFR was then analyzed separately in subgroups with preserved and reduced LVEF. In patients with LVEF ≥ 40%, the difference in eGFR expanded with time and eGFR of sacubitril/valsartan group was 8.2 (95% CI: 3.8–12.6) mL/min/1.72 m^2^ higher than that of valsartan group (*P* < 0.01, Fig. 2A). In patients with LVEF < 40%, the difference of eGFR was not significantly different between sacubitril/valsartan group and valsartan group (Fig. 2B). These findings suggested that sacubitril/valsartan exhibited renal protective effect in patients with HFpEF.

### Differential effect of sacubitril/valsartan on eGFR relative to valsartan in subgroups stratified by baseline eGFR

**Figure 3:**
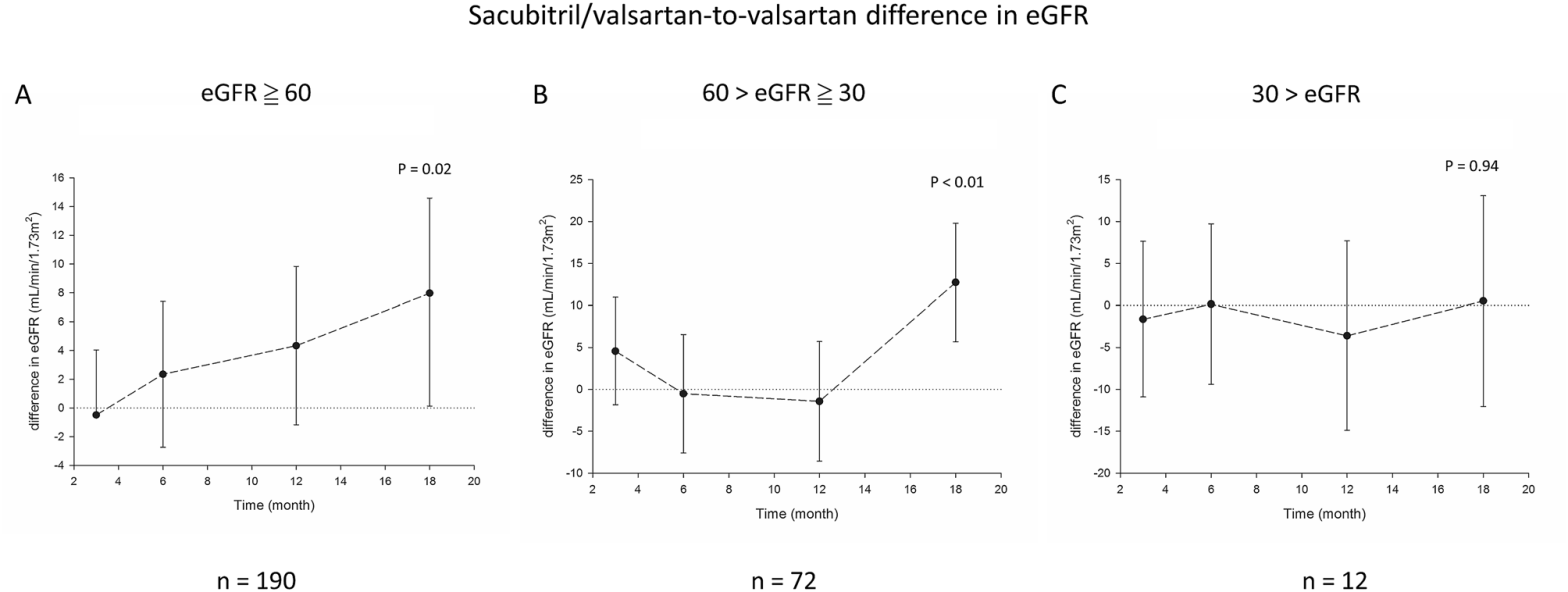
Difference of eGFR between patients on sacubitril/valsartan and valsartan stratified by baseline eGFR. (**A**) eGFR ≥ 60 (**B**) 60 > eGFR ≥ 30. (**C**) 30 > eGFR. eGFR, estimated glomerular filtration rate. *P* value calculated by generalized estimating equation.

In order to investigate the differential effects of sacubitril/valsartan on eGFR in patients with different degree of renal impairment, patients were divided into three subgroups according to baseline eGFR: eGFR ≥ 60 mL/min/1.73 m^2^, eGFR of < 60 to ≥ 30 mL/min/1.73 m^2^, and eGFR < 30 mL/min/1.73 m^2^. In patients with eGFR ≥ 60 mL/min/1.73 m^2^, the difference between the two groups expanded with time and was significant at 18 months, showing that sacubitril/valsartan group had eGFR of 8.0 (95% CI: 1.3–14.6) mL/min/1.73 m^2^ significantly higher than that of valsartan group (*P* = 0.02, Fig. 3A). In patients with eGFR of < 60 to ≥ 30 mL/min/1.73 m^2^, sacubitril/valsartan group had eGFR of 12.7 (95% CI: 5.7–19.8) mL/min/1.73 m^2^ significantly higher than that of valsartan group (*P* < 0.01, Fig. 3B) at 18 months, but exhibited a fluctuated pattern during the follow-up period. In patients eGFR < 30 mL/min/1.73 m^2^, the difference in eGFR was insignificant between the two groups (*P* = 0.94, Fig. 3C). These findings suggested that renal protective effect of sacubitril/valsartan was significant in patients with eGFR ≥ 60 mL/min/1.73 m^2^. In patients with eGFR of < 60 to ≥ 30 mL/min/1.73 m^2^, renal protective effect of sacubitril/valsartan may be less certain in a longer period.

### Effect of sacubitril/valsartan on the risk for renal function decline

The definition of renal function decline was reduction of eGFR ≥ 20% compared to baseline values. In this part of analysis, all demographics, laboratory variables, and medications were evaluated by univariate Cox proportional regression and those with univariate *P* value < 0.05 were included into the multivariate Cox proportional regression model for renal function decline. Among all available demographic and laboratory variables, age and hemoglobin level sufficed the significance to be included into multivariate model (Supplemental Table 2). Among available medications, use of β-blockers, statins, and sacubitril/valsartan (with reference to valsartan use) suffice the significance to be included (Supplemental Table 3). However, as use of β-blockers and statins were highly correlated to use of sacubitril/valsartan (as shown in Table 3), these two medications were not included into the multivariate model to avoid multicollinearity. The final multivariate Cox proportional regression model included age, hemoglobin level, and sacubitril/valsartan or valsartan group. It showed that sacubitril/valsartan group had significantly reduced risk for renal function decline compared to valsartan group (Table 4).

**Table 4:** Risk for function decline by multivariate Cox proportional regression.

Character	HR	95% CI	*P* value
Age, per 10 years increment	1.2	0.9–1.5	0.210
Hemoglobin, per 1 g/dL increment	0.9	0.8–1.0	0.080
Sacubitril/valsartan group (reference: valsartan group)	0.5	0.3–0.9	0.020

*HR* hazard ratio, *CI* confidence interval.

### Effect of sacubitril/valsartan on mortality and HF-related hospitalization

**Figure 4:**
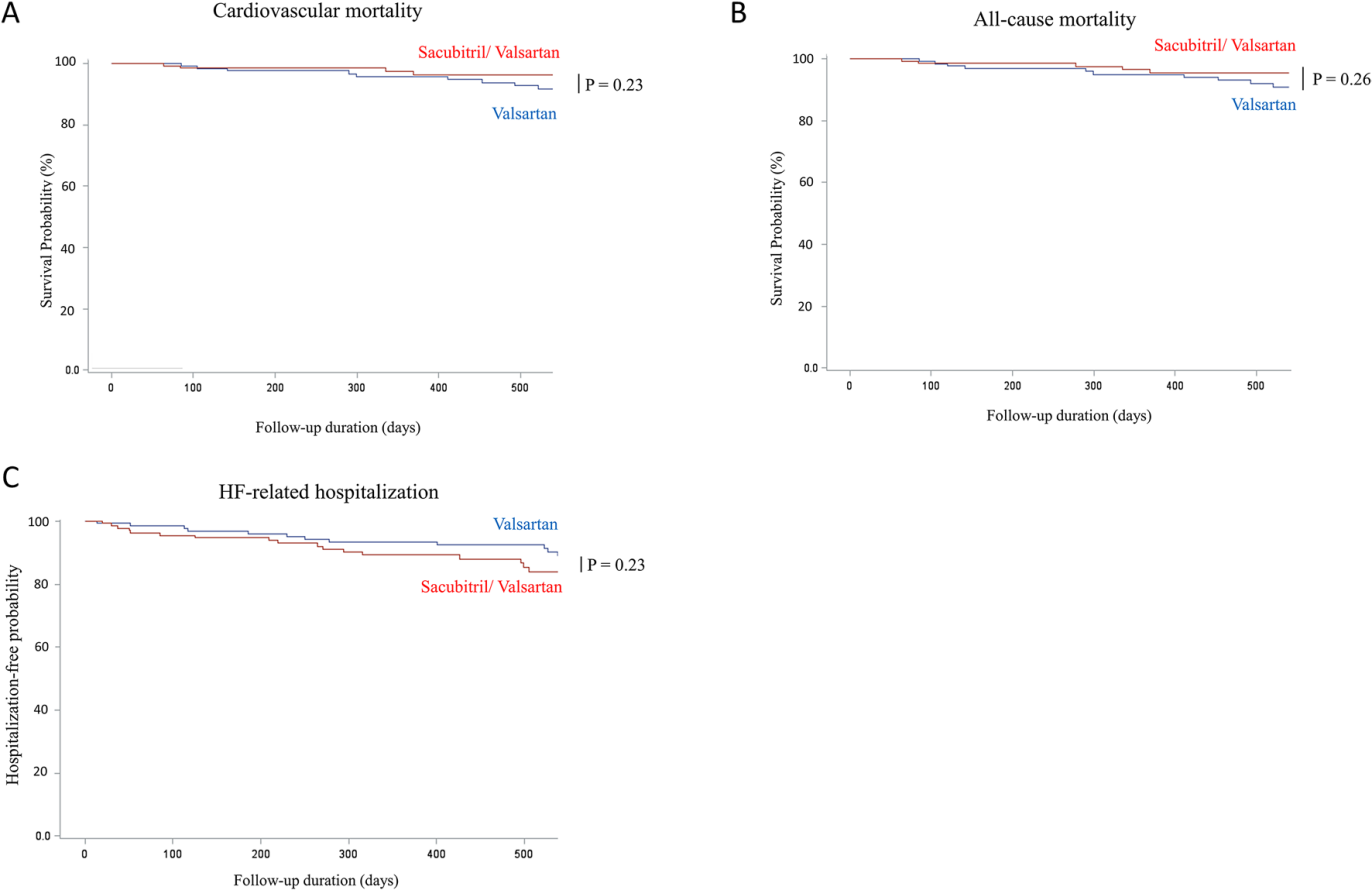
Kaplan–Meier curve for cardiovascular outcomes. (**A**) cardiovascular mortality, (**B**) all-cause mortality, and (**C**) heart failure-related hospitalization. *P* value calculated by log-rank test.

Overall, there were 21 all-cause death and 17 cardiovascular death at the end of study. During the study period, 42 patients had HF-related hospitalization (Table 5). The risk for cardiovascular mortality, all-cause mortality, and HF-related hospitalization were not significantly different between sacubitril/valsartan group and valsartan group (Fig. 4A–C). As stated previously, despite that the two groups had been matched for LVEF, regarding higher AFib, previous HF-related hospitalization, and different medication profile, patients of sacubitril/valsartan group may have higher severity of HF compared with patients of valsartan group. As a result, although the risk for mortality was not significantly different between the two groups, the result implied beneficial effect of sacubitril/valsartan in HF patients, regarding the reduction of mortality and hospitalization rate.

**Table 5:** Mortality and HF-related hospitalization of the cohort.

Outcome variables	Total n = 274	Sacubitril/valsartan n = 137	Valsartan n = 137
Cardiovascular death, n (%)	17 (6.20%)	4 (2.92%)	13 (9.49%)
All-cause death, n (%)	21 (7.66%)	5 (3.65%)	16 (11.68%)
HF-related hospitalization, n (%)	42 (15.33%)	25 (18.25%)	17 (12.41%)

*HF* heart failure.

### Falsification analysis

The falsification outcomes analysis was performed by using Kaplan–Meier curve with log-rank test. All falsification outcomes showed no difference between the sacubitril/valsartan group and valsartan group, suggesting low likelihood of bias effect (Supplemental Fig. 3).

## Discussion

In summary, the present study included two groups of HF patients on sacubitril/valsartan or valsartan, which were matched by gender, age, baseline eGFR and LVEF. After 18 months of follow-up period, sacubitril/valsartan groups had significantly lower eGFR decrement, specifically in subgroups with HFpEF or baseline eGFR ≥ 60 mL/min/1.73 m^2^. In addition, sacubitril/valsartan group had less renal function decline events. Overall, our findings suggested independent renal protective effect of neprilysin inhibition. Regarding the risk for mortality and HF-related hospitalization, there was no significant difference between the two groups, which may be explained by that the majority of patients in this study had preserved EF.

In a randomized double-blind trial conducted by the UK HARP-III Collaborative Group, Haynes et al. compared the difference of eGFR between patients on sacubitril/valsartan and on irbesartan. This study enrolled CKD patients with few cases of HF and baseline eGFR was 20 to 60 mL/min/1.73 m^2^. eGFR measured at 12 months were not significantly different between the two groups^26^. Three factors may contribute to negative result in this study. First, around 40% of patients in this study had eGFR of < 30 mL/min/1.73 m^2^, who were less sensitive to renal protective effect of sacubitril/valsartan as shown in our study. On the contrary, the majority of patients included in the present study had eGFR of ≥ 60 mL/min/1.73 m^2^, who were more sensitive to renal protective effect of sacubitril/valsartan. Second, 12 months of follow-up may be insufficient to observe a significant difference in renal function decline, as the significant difference in eGFR occurred at 18 months in the present study. For the same reason, similar trials conducted for shorter periods revealed negative results on renal outcomes^16,17^. Third, only 3–4% of the participants in this previous study had HF. On the contrary, the present study exclusively enrolled patients with HF (HFpEF, 77.4%; HFrEF, 22.6%). This difference in HF status may contribute to the different renal results between the two studies. Also, the different renal results suggested that that the renal protective effect of neprilysin inhibition may be restricted to patients with HF, especially in those with HFpEF.

Whether neprilysin inhibition independently has renal protective effect had been an issue of debate. In a secondary analysis of the PARADIGM-HF trial enrolling 8399 patients with 44 months of follow-up period, Zile’s group concluded that the addition of neprelysin inhibition attenuated renal function decline in HF patients with DM^27^. Nonetheless, the control group of PARAGIGM-HF trials were treated with enalapril and the renal outcome of this study may be biased by the difference between valsartan and enalapril rather than the effect of neprilysin inhibition. The result of the present study, in which the control group was on valsartan, may serve as a supporting information for the renal protective effect of neprilysin inhibition.

The results of the present study showed no significant difference between sacubitril/valsartan and valsartan regarding cardiovascular mortality, all-cause mortality and HF-related hospitalization. One explanation to the result may be that sacubitril/valsartan group had higher severity of HF at baseline than valsartan group in this study, as previously stated. A second explanation may be that our study included too many patients with preserved EF. In PARADIGM-HF trial that recruited patients with HFrEF, sacubitril/valsartan significantly reduced the risk of death and of hospitalization for HF^15^. Nonetheless, in PARAGON-HF trial that enrolled patients with HFpEF, sacubitril/valsartan did not significantly improve mortality or hospitalization of any cause^28^. According to the results of these two trials, the beneficial effect of sacubitril/valsartan on HF is restricted to patients with HFrEF. This may also explain the finding on cardiovascular mortality, all-cause mortality and HF-related hospitalization in the present study.

The present study showed that sacubitril/valsartan had renal protective specifically in patients with HFpEF. In PARAGON-HF trial, which investigated the effect of sacubitril/valsartan versus valsartan in patients with HFpEF, mean baseline eGFR was 62–63 mL/min/1.73 m^2^ and the renal composite outcome was the occurrence renal decline ≥ 50%, development of ESRD, death due to renal failure. In this trial, the occurrence of renal composite outcome was not significantly different between the two groups. Nonetheless, adverse renal event (elevated serum creatinine ≥ 2.0 mg/dL) was significantly fewer in the sacubitril/valsartan group^28^. The results of this trial along with our finding suggested that sacubitril/valsartan may have renal protective effect in patients with HFpEF.

One of the limitations of the present study was the retrospective design that restricted us to control all HF-related factors. Although we matched the patients for age, gender, LVEF, and eGFR, severity of HF classed by New York Heart Association Function Class was absent. In our cohort, sacubitril/valsartan group had more patients with AFib and previous HF-related hospitalization which implied higher severity of HF. Nonetheless, despite of higher HF severity in sacubitril/valsartan group, our result demonstrated superior renal outcomes in this group, suggesting its renal protective effect in HF patients. A second limitation was that albuminuria was not included in the outcome variables due to a large part of missing values, which had been a major outcome in most randomized controlled trials. This limitation also made us unable to define CKD stage 1 and 2 in this study. A third limitation was the small case number, which restricted statistical power. A fourth limitation was the single ethnicity in this study, which restricted the validity of applicating our result to non-Asian patients. Besides, echocardiographic measurement of E/E’ was absent in our data, which limited our estimation of diastolic dysfunction.

In conclusion, this retrospective cohort study showed renal protective effect of neprilysin inhibition in HF patients, specifically in subgroups with HFpEF or baseline eGFR ≥ 60 mL/min/1.73 m^2^. The findings of the present study suggested that sacubitril/valsartan may be an optimal treatment for HF patients with eGFR ≥ 60 mL/min/1.73 m^2^. To confirm the findings of the present study, a clinical trial that enrolls patients susceptible to its renal protective effect is required in future.

## Supplementary Information

Supplementary Information 1.
