# Supplementary material for: Renal protective effect of sacubitril/valsartan in patients with heart failure
Source: Sci Rep. 2021 Feb 25;11:4593. doi: 10.1038/s41598-021-84118-8 (PMC7907094; doi:10.1038/s41598-021-84118-8)
Supplement: Supplementary file 1 — Supplementary Information 1. [file 41598_2021_84118_MOESM1_ESM.docx]

**Renal protective effect of Sacubitril/Valsartan in patients with heart failure – Supplemental File**

Hui-Ling Hsieh, MSN^1,2^, Chun-You Chen, MD^3^¶, Cheng-Hsien Chen, PhD^1,4,5^¶, Shih-Chang Hsu, MS, MD^6,7^, Wen-Cheng Huang, MD^6,7,8^, Yuh-Mou Sue, MD^1,5^, Feng-Yen Lin, PhD^5,9^, Chun-Ming Shih, MD, PhD^5,9^, Yue-Cune Chang, PhD^10^, Po-Hsun Huang, MD, PhD^11,12,13^‡, Chung-Te Liu, MD^1,5,8^‡*

^1^Division of Nephrology, Department of Internal Medicine, Wan Fang Hospital, Taipei Medical University, Taipei, Taiwan

^2^Graduate Institute of Medical Science, National Defense Medical Center, Taipei, Taiwan

^3^Department of Radiation Oncology, Wan Fang Hospital, Taipei Medical University, Taipei, Taiwan

^4^Division of Nephrology, Department of Internal Medicine, Shuang Ho Hospital, Taipei Medical University, New Taipei City, Taiwan

^5^Department of Internal Medicine, School of Medicine, College of Medicine, Taipei Medical University, Taiwan.

^6^Emergency Department, Department of Emergency and Critical Medicine, Wan Fang Hospital, Taipei Medical University, Taipei, Taiwan.

^7^Department of Emergency Medicine, School of Medicine, College of Medicine, Taipei Medical University, Taipei, Taiwan

^8^Graduate Institute of Clinical Medicine, College of Medicine, Taipei Medical University, Taipei, Taiwan

^9^Division of Cardiology and Cardiovascular Research Center, Department of Internal Medicine, Taipei Medical University Hospital, Taipei, Taiwan

^10^Department of Mathematics, Tamkang University, Taipei, Taiwan

^11^Division of Cardiology, Department of Medicine, Taipei Veterans General Hospital, Taipei, Taiwan

^12^Cardiovascular Research Center, National Yang-Ming University, Taipei, Taiwan

^13^Institute of Clinical Medicine, National Yang-Ming University, Taipei, Taiwan

¶ These authors contributed equally to this work.

‡ These authors contributed equally to this work.

***Corresponding author**:

Chung-te Liu

Division of Nephrology, Department of Internal Medicine, Wan Fang Hospital, Taipei Medical University, Taipei, Taiwan

Fax number: 886-2930-2448

Telephone number:886-970746583

E-mail: 96320@w.tmu.edu.tw

**Table of contents**

**Supplemental Table 1. Baseline characteristics before and after matching**

**Supplemental table 2. Baseline characteristics and risk for renal function decline**

**Supplemental table 3. Baseline medications and risk for renal function decline**

**Supplemental Figure 1. Flow diagram of the study**

**Supplemental Figure 2. Change in eGFR with time in the study cohort**

**Supplemental Figure 3. Kaplan-Meier curve for falsification analysis. A. Cancer Events, B. Pneumonia Events, and C. Fractures Events.**

| **Supplemental Table 1. Baseline characteristics before and after matching** | | | | | |
| --- | --- | --- | --- | --- | --- |
| Characteristics |  | Before match | After match | P value | Standardized difference |
| sacubitril/valsartan |  | n = 221 | n = 137 |  |  |
| Male, n (%) |  | 145 (65.6) | 90 (65.7) | 0.987 | n/a |
| Age, years |  | 70.5±15.2 | 72.6±14.9 | 0.196 | 0.140 |
| LVEF, % |  | 46.1±16.7 | 52.6±15.7 | <0.001 | 0.401 |
| eGFR, mL/min/1.73m^2^ |  | 66.0±28.7 | 70.9±24.6 | 0.100 | 0.184 |
|  |  |  |  |  |  |
| valsartan |  | n = 14030 | n = 137 |  |  |
| Male, n (%) |  | 8067 (57.5) | 90 (65.7) | 0.053 | n/a |
| Age, years |  | 67.0±12.7 | 72.6±14.9 | <0.001 | 0.406 |
| LVEF, % |  | 69.4±57.3 | 55.3±15.6 | <0.001 | 0.387 |
| eGFR, mL/min/1.73m^2^ |  | 80.1±24.1 | 70.9±24.6 | <0.001 | 0.378 |

n/a, not applicable.

LVEF, left ventricular ejection fraction; eGFR, estimated glomerular filtration rate.

| **Supplemental table 2. Baseline characteristics and risk for renal function decline*** | | | |
| --- | --- | --- | --- |
| Characters | HR | 95% CI | P value |
| Male | 1.8 | 0.4-1.3 | 0.299 |
| Age, per 10 years increment | 1.3 | 1.1-1.6 | 0.009 |
| DM | 1.6 | 0.9-2.6 | 0.103 |
| AFib | 1.1 | 0.6-2.1 | 0.809 |
| HF-related hospitalization | 0.6 | 0.3-1.4 | 0.583 |
| LVEF, per 10% increment | 0.9 | 0.8-1.1 | 0.442 |
| eGFR, per 10 mL/min/1.73m^2^ increment | 0.9 | 0.8-1.0 | 0.062 |
| AST, per 10 U/L increment | 1.1 | 0.8-1.3 | 0.661 |
| ALT, per 10 U/L increment | 0.8 | 0.7-1.1 | 0.199 |
| Hemoglobin, per 1 g/dL increment | 0.8 | 0.7-0.9 | 0.014 |
| K, mmol/L | 0.8 | 0.5-1.3 | 0.415 |
| WBC, per 10^3^/uL increment | 1.0 | 0.9-1.1 | 0.719 |

*by univariate Cox proportional regression model

HR, hazard ratio; CI, confidence interval; DM, diabetes mellitus, Afib, atrial fibrillation; HF, heart failure ; LVEF, left ventricular ejection fraction; eGFR, estimated glomerular filtration rate; AST, aspartate aminotransferase; ALT, alanine aminotransferase; WBC, white blood cell.

| **Supplemental table 3. Baseline medications and risk for renal function decline*** | | | |
| --- | --- | --- | --- |
| Characters | HR | 95% CI | P value |
| Beta blockers | 0.5 | 0.3-0.8 | 0.007 |
| Dihydropiridine CCB | 1.4 | 0.7-2.6 | 0.299 |
| Non-dihydropiridine CCB | 0.5 | 0.1-3.2 | 0.423 |
| NTG | 1.1 | 0.5-2.9 | 0.770 |
| Amiodarone | 0.8 | 0.3-2.1 | 0.600 |
| Aspirin | 0.6 | 0.3-1.2 | 0.565 |
| Clopidogrel | 1.0 | 0.6-1.9 | 0.926 |
| Rivaroxaban | 0.5 | 0.1-1.5 | 0.193 |
| Warfarin | 0.7 | 0.1-4.9 | 0.700 |
| Febuxostat | 0.6 | 0.2-1.5 | 0.278 |
| Fibrates | 0.9 | 0.2-3.7 | 0.894 |
| Statin | 0.6 | 0.3-0.9 | 0.048 |
| Sacubitril/valsartan group (reference: valsartan group) | 0.5 | 0.3-0.8 | 0.008 |

*by univariate Cox proportional regression model

HR, hazard ratio; CI, confidence interval; CCB, calcium channel blocker; NTG, nitroglycerin.


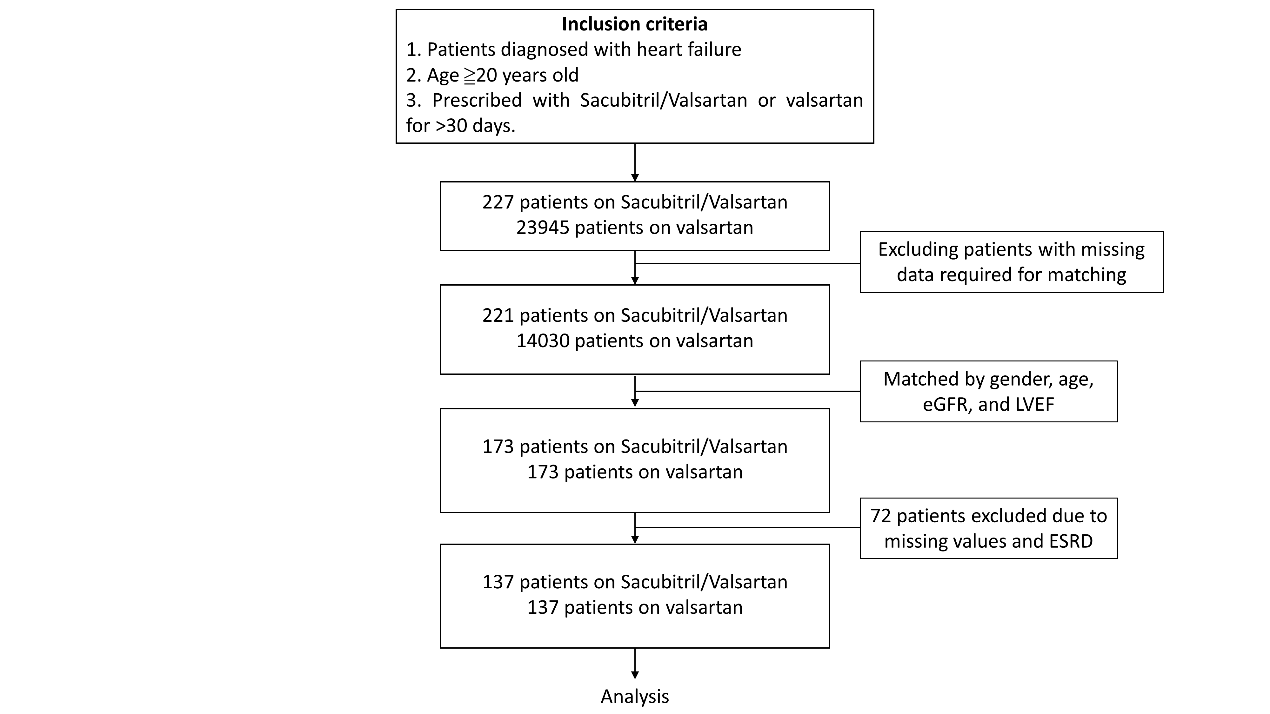


**Supplemental Figure 1. Flow diagram of the study.**

eGFR, estimated glomerular filtration rate; LVEF, left ventricular ejection fraction; ESRD, end-stage renal disease.


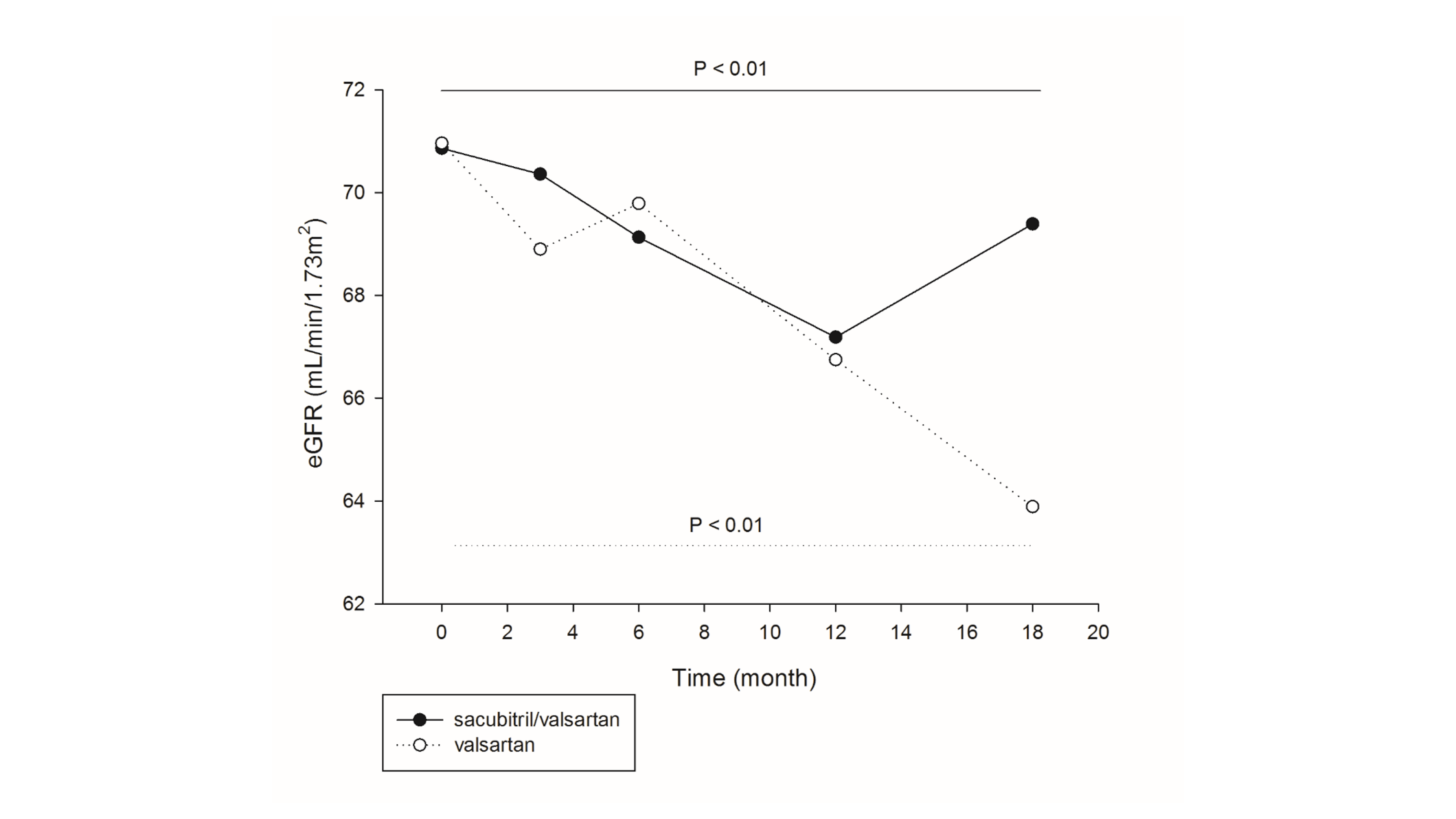


**Supplemental Figure 2. Change in eGFR with time in the study cohort.**

eGFR, estimated glomerular filtration rate.


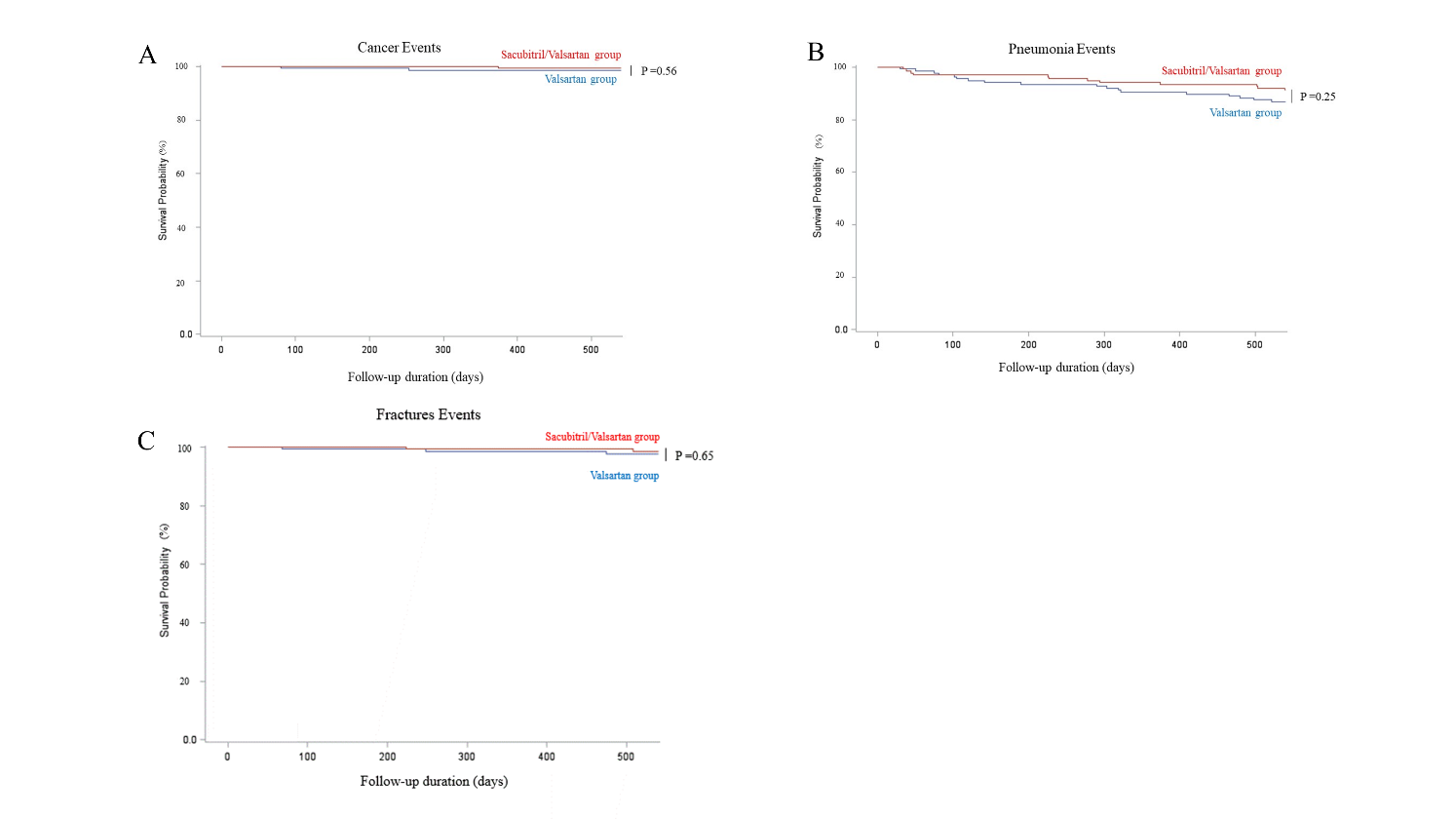


**Supplemental Figure 3. Kaplan-Meier curve for falsification analysis. a. Cancer Events, b. Pneumonia Events, and c. Fractures Events.**

P value calculated by log-rank test.
